# Multichannel Transcranial Direct Current Stimulation Combined With Treadmill Gait Training in Patients With Parkinson's Disease: A Pilot Study

**DOI:** 10.3389/fneur.2022.804206

**Published:** 2022-03-16

**Authors:** Yoonju Na, Jinuk Kim, Su-Hyun Lee, Jihye Kim, Jungsoo Lee, Se Young Shin, Won Hyuk Chang, Jin Whan Cho, Yun-Hee Kim

**Affiliations:** ^1^Department of Physical and Rehabilitation Medicine, Center for Prevention and Rehabilitation, Heart Vascular Stroke Institute, Samsung Medical Center, Sungkyunkwan University School of Medicine, Seoul, South Korea; ^2^Department of Health Sciences and Technology, SAIHST, Sungkyunkwan University, Seoul, South Korea; ^3^Department of Medical IT Convergence Engineering, Kumoh National Institute of Technology, Gumi, South Korea; ^4^Department of Neurology, Samsung Medical Center, Sungkyunkwan University School of Medicine, Seoul, South Korea; ^5^Department of Medical Device Management & Research, Department of Digital Health, SAIHST, Sungkyunkwan University, Seoul, South Korea

**Keywords:** Parkinson's disease, gait training, multichannel transcranial direct current stimulation (tDCS), non-invasive brain stimulation (NIBS), neurorehabilitation

## Abstract

**Background:**

Gait problems are critical impairments in Parkinson's disease (PD) and are related to increased risk of fall and negatively impact activities of daily life. Transcranial direct current stimulation (tDCS) is a non-invasive brain stimulation technique that can modify the cortical excitability of gait-related brain regions. In this study, we investigated whether multichannel tDCS with simultaneous treadmill gait training could improve gait in PD.

**Methods:**

Twenty-four patients with PD were assigned randomly to a real or sham tDCS group. Before intervention, one patient of the real tDCS group was dropped out, leaving 23 patients to be analyzed in this study. Each patient underwent 30 min of treadmill gait training for 10 sessions over four consecutive weeks. Multichannel 4x1 tDCS was applied using five 6-cm-diameter round electrodes. One anode was placed on the CZ, and four cathodes were positioned symmetrically over the FZ, C5, C6, and PZ. Anodal tDCS (2mA) and sham tDCS were delivered for 20 min. The secondary outcomes were gait performance, as measured by the timed up and go test (TUG) and freezing of gait questionnaire (FOG-Q), and balance was assessed using the dynamic gait index (DGI), Berg balance scale (BBS), and functional reach test (FRT). Motor and non-motor performance of patients with PD were assessed using the Movement Disorder Society-sponsored revision of the Unified Parkinson's Disease Rating Scale (MDS-UPDRS). Participants were assessed before the intervention, immediately after the intervention, and 4 weeks after completion of the intervention.

**Results:**

The real tDCS group showed a significant improvement in the 10-m walk test, but the sham group did not. Among the secondary outcome measures, MDS-UPDRS part II, TUG, and BBS were improved only in the real tDCS group. Particularly, MDS-UPDRS part II showed a significant group^*^time interaction effect, indicating that real tDCS demonstrated a better effect on the activities of daily living patients with PD.

**Conclusions:**

The results of this pilot study suggest that multichannel tDCS applied on the leg motor cortex during treadmill gait training is a safe and effective means to improve gait velocity in patients with PD. Additional rigorous, large-sample, multicenter, randomized controlled trials are needed to confirm the effect of tDCS as a therapeutic adjunct for gait rehabilitation of patients with PD.

## Introduction

Parkinson's disease (PD) is a progressive neurodegenerative disease characterized by four major motor signs: resting tremor, rigidity, bradykinesia, and postural instability (1). Gait difficulties and balancing issues are disabling problems in many patients with PD, with different contributing factors, such as freezing of gait (FOG), festination, shuffling steps, and progressive loss of postural reflexes.

In advanced PD, difficulties of gait and postural control, bradykinesia, cognitive impairment, and non-motor symptoms, refractory to conventional treatment, pose therapeutic challenges. When the disease progresses, gait and balance problems can worsen and underlie the risk of fall, leading to hip fracture in later stages of PD. Patients with PD have a 2-fold higher mortality related to fall injuries compared to individuals without PD (2). Gait disturbances, such as FOG and falls, are often resistant to pharmacological interventions, and rehabilitation programs often are not able to limit the progression of PD (3).

The success of deep brain stimulation (DBS) and advances in the understanding of the pathophysiology of PD have increased interest in non-invasive brain stimulation (NIBS) techniques as alternative therapeutic tools. Benninger et al. (4) first reported transcranial direct current stimulation (tDCS) effect on patients with PD. Patients with PD who did not take medication underwent anodal tDCS applied to the primary motor cortex (M1_leg_), and they reported better improvement in bradykinesia compared to patients receiving sham stimulation (5). The composite of the Movement Disorder Society-sponsored revision of the Unified Parkinson's Disease Rating Scale (MDS-UPDRS) bradykinesia score indicated improvement with anodal tDCS in the off-medication state. To date, tDCS has been explored for treatment in patients with PD by virtue of its ability to influence motor and non-motor symptoms (6). According to a systematic review (6), multiple studies have assessed the efficacy of tDCS as a treatment for gait symptoms in patients with PD. tDCS over motor areas showed promising results, with seven of the 10 studies showing a positive effect of motor area stimulation on gait. Studies also have reported that DLPFC stimulation improved gait speed in PD.

In a real clinical setting, stimulating the M1_leg_ using tDCS involves a challenge due to its depth and orientation within the interhemispheric fissure. Conventional tDCS produces a wide-spread electric field with low spatial specificity, reaching target areas of the brain with low density. To increase the density and focality of current to the target area, multi-tDCS montages with multiple configurations were used in this study (7). The most used multi-tDCS montage is the 4 × 1 ring configuration, which consists of one active electrode placed on the area of interest and four reference electrodes surrounding it. A previous study offered a clue that a 4 × 1 multi-tDCS was capable of stimulating the deep interhemispheric cortical area of the dorsal anterior cingulate cortex (ACC) (8). The electric current delivered is constrained and localized within the reference electrodes. Multi-tDCS is an approach capable of efficiently targeting distributed brain networks to facilitate beneficial neuroplasticity and functional connectivity (9).

A systematic review (10) defined physical therapy/exercise for patients with PD as activities focusing on muscle strengthening and enhancing aerobic capacity, balance, gait, and functional mobility. Most trials and review articles demonstrated that physical therapy/exercise improved gait performance in terms of speed, stride, or step-length, and increased the walking capacity as measured by the 6-min walk test (11, 12). According to animal research (13), physical activity manifests neurorestorative and neuroprotective effects. Exercise on a treadmill can raise the level of neurotrophins in the striatum in rat models with PD. The presence of exercise-induced neuroplasticity in PD is further supported by human studies (14). Treadmill training led to the enhancement of corticomotor excitability, which was associated with improved gait parameters. In our study, we posited that the combination of tDCS and treadmill training would intensify the effect of physical therapy for patients with PD.

In the present double-blind, randomized, sham-controlled study, we aimed to confirm that patients who undergo multi-tDCS to the M1_leg_ and treadmill training would show better gait performance including speed, freezing symptom, and balance than the control group that only undergoes treadmill training. We also investigated whether such stimulation is safe concurrent with treadmill training. We tried to determine the benefits to balance, FOG, disease severity, and non-motor aspects of patients with PD beyond those of the current therapy, such as medications.

## Methods

### Participants

Participants included patients who were diagnosed with PD based on published criteria (15).

The clinical trial protocol and consent form were reviewed and approved by the Korean Food and Drug Administration (No. 1159) and the Institutional Review Board at Samsung Medical Center, Seoul, Republic of Korea (IRB-2020-09-093). The trial was registered with ClinicalTrials.gov (NCT04591236). Written informed consent was obtained from all the patients. Inclusion criteria were patients aged 50–75 years with Modified Hoehn and Yahr stages of 1–4 while on medication. Doses and types of medication were not changed during the study period. Subjects were excluded if they had a history of severe major neurological diseases such as stroke or traumatic brain injury, psychiatric disease such as schizophrenia, or severe cognitive impairment, as assessed by the Korean mini-mental state examination (K-MMSE), with a score less than 10. Subjects with contraindications of tDCS such as intracranial metallic implants, history of seizure, pregnancy, implanted electrical devices such as pacemakers, and skin problems on the scalp that might be worsened during tDCS were also excluded from the study. This study is a pilot study, so the sample size was not calculated statistically.

**Figure 1:**
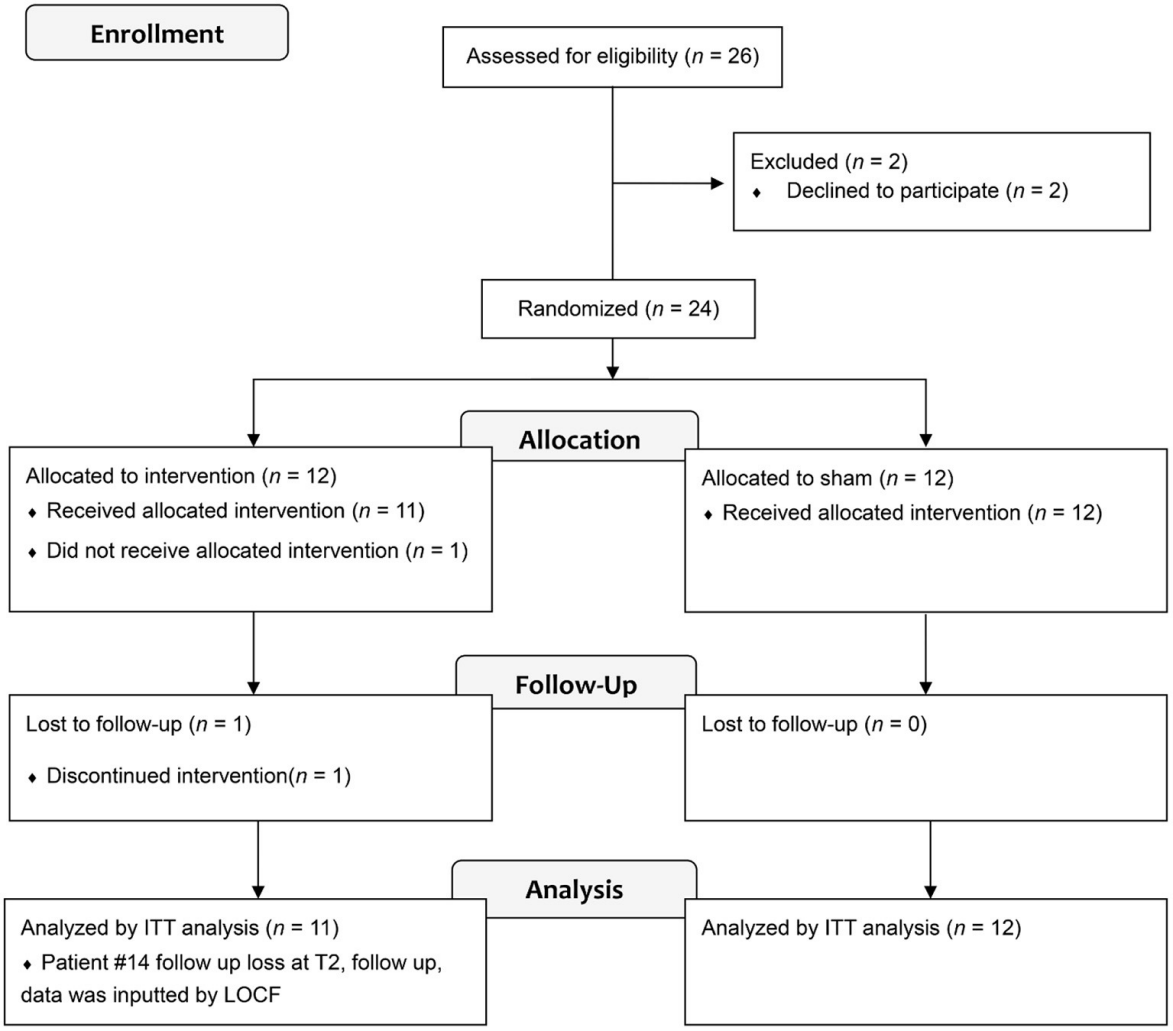
Flow diagram of the study.

We assessed 26 patients for eligibility, and two declined to participate (Figure 1). Finally, we recruited 24 patients and randomized them into either the real tDCS (r-tDCS) intervention with treadmill gait training group (*n* = 12, 8 women, mean age 63.73 ± 6.57) or the sham tDCS (s-tDCS) intervention with treadmill gait training group (*n* = 12, 7 women, mean age 65.08 ± 6.46). The two groups conducted the same treadmill gait training during this study. One patient in the r-tDCS group was lost to follow up. This study is a pilot study, and the sample size was not calculated statistically. We referred to the current rules of thumb for a two-arm pilot trial sample (16). Dopaminergic regimens were maintained without change throughout the study. Patients were assessed during the “on” medication state for all study evaluations. We documented the data of levodopa equivalent dose (LED) in Table 1. There was no difference between the groups. All patients were randomized using a table of random numbers by a researcher who was not otherwise part of this study. One patient in the r-tDCS group dropped out during follow-up, and associated data were input by the last observation carried forward (LOCF). The demographic and clinical findings of patients in the r-tDCS (*n* = 11) and s-tDCS (*n* = 12) intervention groups were comparable (Table 1). Written informed consent was obtained from all the participants before inclusion in the study. Ethical approval was obtained from the Institutional Review Board (IRB) of Samsung Medical Center, Seoul, Republic of Korea.

**Table 1:** Baseline characteristics of the study population.

	**r-tDCS group**	**s-tDCS group**	***p* value**
	**(*n* = 11)**	**(*n* = 12)**
Age, years (mean ± SD)	63.73 ± 6.57	65.08 ± 6.46	0.62^a^
Sex, F:M	8:3	5:7	0.47^b^
Height, cm (mean ± SD)	157.04 ± 7.54	158.55 ± 6.63	0.61^b^
Body weight, kg (mean ± SD)	58.05	65.85 ± 11.23	0.14^a^
BMI (mean ± SD)	23.30± 3.22	26.24 ± 4.57	0.09^a^
Hoehn and Yahr, (median and range)	1, range 1~3	2, range 1~3	0.78^b^
LED, mg (mean ± SD)	564.91 ± 350.79	766.58 ± 267.35	0.13^a^
10MWT, m/s (mean ± SD)	1.11 ± 0.19	1.15 ± 0.26	0.68^a^
TUG, sec (mean ± SD)	10.35± 2.85	9.82± 1.97	0.60^a^
FRT, cm (mean ± SD)	20.14 ± 7.34	17.77± 6.24	0.41^a^
FOG-Q (mean ± SD)	7.27 ± 2.28	8.72± 3.52	0.25^a^
FOG-Q #3 (*n*,%)			0.15^b^
Point 0	0	0	
Point 1	6 (54.5)	4 (33.3)	
Point 2	5 (45.5)	6 (50.0)	
Point 3	0	2 (16.7)	
Point 4	0	0	
BBS (mean ± SD)	44.18 ± 3.68	40.75 ± 7.35	0.17^a^
DGI (mean ± SD)	18.18 ± 4.58	15.50 ± 3.63	0.95^c^
K-MMSE (mean ± SD)	28.00 ± 1.48	28.42 ± 1.24	0.47^a^
GDS (mean ± SD)	5.55 ± 2.50	7.08 ± 4.29	0.31^c^
MDS-UPDRS Part 1 (mean ± SD)	11.45 ± 4.55	12.75 ± 5.26	0.54^a^
MDS-UPDRS Part 2 (mean ± SD)	9.45 ± 3.64	12.58 ± 6.33	0.44^c^
MDS-UPDRS Part 3 (mean ± SD)	33.64 ± 16.06	34.50 ± 12.67	0.89^a^
MDS-UPDRS Part 4 (mean ± SD)	1.09 ± 2.12	3.50 ± 4.91	0.14^c^
MEP, rMT of Lt leg (mean ± SD)	36.45 ± 6.19	40.25 ± 9.35	0.27^a^
MEP, rMT of Rt leg (mean ± SD)	41.09 ± 9.03	43.17 ± 7.00	0.54^a^
MEP, AMP of Lt leg (mean ± SD)	525.18 ± 212.37	576.08 ± 360.24	0.68^a^
MEP, AMP of Rt leg (mean ± SD)	749.91 ± 423.43	621.50 ± 503.29	0.18^c^

*tDCS, Transcranial Direct Current Stimulation; BMI, Body Mass Index; LED, Levodopa Equivalent Dose; TUG, Timed Up and Go Test; FRT, Functional Reach Test; FOG-Q, Freezing of Gait Questionnaire; BBS, Berg Balance Scale; DGI, Dynamic Gait Index; K-MMSE, Korean Mini-Mental State Examination; GDS, Geriatric Depression Scale; MDS-UPDRS, Movement Disorder Society-sponsored revision of the Unified Parkinson's Disease Rating Scale; MEP, Motor Evoked Potential; rMT, Resting Motor Threshold; AMP, Amplitude*.

a *Independent t-test;*

b *Chi-square test;*

c *Mann-Whitney test*.

### Study Design

A prospective, analytical, double-blinded, randomized, sham-controlled clinical trial was carried out. This study was double-blinded for researchers and patients. Among researchers, the two researchers who measured gait performances of subjects were not involved in the interventions and were unaware of the assigned groups. This is a pilot study for investigating the safety and effectiveness of multichannel tDCS applied simultaneously with treadmill gait training for patients with PD.

Participants were randomized to (1) treadmill with real tDCS and (2) treadmill with sham tDCS. The trial duration was 4 weeks, with a total of 10 sessions of intervention, and endpoint assessment was performed 4 weeks after the end of the intervention period. Patients in Group I (r-tDCS) received tDCS for the first 20 min of a 30-min period of treadmill gait training. Patients in Group II (s-tDCS) received sham tDCS during the same physical training. Outcomes were recorded before intervention (T0), immediately after intervention (T1), and 4 weeks after intervention (T2).

### Treadmill Gait Training

For treadmill gait training, the GAT SYSTEM Pro (Cybermedia Inc., Korea) treadmill was used. The velocity can be adjusted in increments of 0.1 km/h through a display embedded in the treadmill. The velocity range is 0–8 km/h. Treadmill gait training was performed at a self-selected speed for 30 min. Self-selected treadmill gait speeds of both the groups in each section are reported in Supplementary Table 1. To perform timed tests of gait after the intervention, the patients determined a comfortable treadmill speed before every session during 2 min of warm-up walking. After 3 min of rest, the intervention began. During treadmill training, we used a harness to protect against falls, but it was not supporting the body weight. While the patient was walking on the treadmill, safety personnel were by their side.

### tDCS Intervention

tDCS was administered using a battery-driven stimulator (YDS-401B, ybrain Inc., Korea) that delivers direct electric current through saline-soaked sponge electrodes. For each real tDCS session, we applied a direct anodal current of 2 mA through a rubber pad with a sponge-inserted electrode (surface 28.26 cm^2^, diameter 6 cm, current density 0.071 mA/cm^2^) through an anode placed over the CZ (M1_leg_ area target region) of the 10–20 international electroencephalographic system. Anodal electrodes were symmetrically located around the CZ. Considering the electric current direction, tDCS intervention was based on bihemispheric montage. tDCS intervention was conducted for 20 min/day at the beginning of a 30-min period of treadmill training. Four cathodes were positioned symmetrically over the FZ, C5, C6, and PZ. In the first 10 s, stimulation was gradually increased to 2 mA, where it was of help, until gradually decreasing in the last 10-s of the session. The ramp-up and ramp-down times were each 10 s. The level of impedance was variable to supply constant current. For safety reasons, the instrument was designed to deactivate the power supply over a certain level of impedance. In the s-tDCS group, we placed the anode and cathodes over the same positions as in the r-tDCS group. The device was turned on without sending current. We set up the devices out of sight of the patients and blinded the investigators. We observed events of fall without injury, fall with hospitalization, hypotension, dizziness, muscle pain, minor injuries, and self-reported adverse effects during training and intervention. We listed possible symptoms and asked patients to respond with “Yes” (if yes, specify the event type) or “No.” At the end of each session, the side effect survey was performed by interviewing patients. We used a documented questionnaire about adverse events and side effects for each patient. The experiment was performed for 10 sessions with intervention (3 weekly sessions for 4 weeks) during an “on” medication period. The study was approved by the Ministry of Food and Drug Safety of the Republic of Korea. They also approved this tDCS device for use in this clinical trial.

### Clinical Assessments

Baseline and follow-up evaluations were performed before the intervention (T0), immediately after 10 sessions (T1), and 4 weeks after completing the intervention (T2). The primary outcome measure was gait speed measured at a comfortable pace. The 10-m walk test (10MWT) was used to determine gait speed as it is a common tool reliable for assessing gait speed in patients with PD (17).

Secondary outcome measures included timed up and go test (TUG), functional reach test (FRT), freezing of gait questionnaire (FOG-Q), Berg balance scale (BBS), and dynamic gait index (DGI). Selective disease-related disabilities, including non-motor aspects, were assessed by the MDS-UPDRS parts II and III. We also measured MDS-UPDRS parts I and IV to investigate additional effects, such as behavior, mood, and motor complication, of our intervention even though these are not gait-related outcomes.

We recorded the MDS-UPDRS to determine different aspects of PD symptoms, gauged the severity and progression of PD, and analyzed each part separately: part I, non-motor aspects of experiences of daily living (6 items assessed by interview and 7 items by self-assessment); part II, motor aspects of experiences of daily living (13 self-assessed items); part III, motor examination (18 items resulting in 33 scores by location and lateralization); and part IV, motor complications (3 items for dyskinesia and 3 for fluctuation) (18). To evaluate mobility, the TUG evaluated the time required to rise from a chair, walk 3 m at a comfortable pace, turn, return to the chair, and sit down (19). We assessed a participant's stability and dynamic balance, referred to as frailty and risk of falling, respectively, by measuring FRT (20). Freezing of gait severity was recorded based on FOG-Q (21). For evaluating balance function during sitting, standing, and changing positions, BBS (22) was measured. Also, DGI (23) was assessed to record not only the ability to maintain balance while walking, but also to walk in the presence of external demands.

### Statistical Analysis

Statistical analyses were performed using the SPSS statistical software (version 27; SPSS, Inc., Chicago, IL, USA). *P* values less than 0.05 were considered significant. The Chi-square test was used to compare non-parametric data among the two groups. The Shapiro–Wilk test was applied to explore the normality of baseline and outcome data. An independent samples *t-*test was used to evaluate the group (r-tDCS vs. s-tDCS) differences in assessment scores between time points (ΔT1-T0 and ΔT2-T0). The Mann–Whitney test was used for DGI and the MDS-UPDRS parts III and IV. Repeated measures analysis of variance (RM-ANOVA) with least significant differences were used to examine the changes in clinical assessment scores across the three time-points.

## Results

Figure 1 shows the flow diagram of the patients. A total of 24 enrolled patients completed the study, but one patient in the r-tDCS group was lost to follow-up after the tDCS intervention was completed. At baseline, neither demographic nor primary and secondary outcome data differed between the groups (Table 1). Regarding changes between T1, T2, and T0, Table 2 shows the mean ± standard error of the mean of each clinical assessment. In this study, no side effects of tDCS or treadmill gait training were reported in any of the 23 patients.

**Table 2:** Differences between gait and balance measures at various time points between groups undergoing r-tDCS or s-tDCS interventions.

**Assessment scores**	**ΔT1-T0**		***p* value**	**ΔT2-T0**		***p* value**
	**r-tDCS**	**s-tDCS**		**r-tDCS**	**s-tDCS**	
10MWT	0.14 ± 0.16	0.06 ± 0.30	0.450	0.24 ± 0.19	0.07 ± 0.26	0.101
TUG	−1.51 ± 1.90	−0.52 ± 2.33	0.279	−2.03 ± 1.86	−0.88 ± 1.93	0.162
FRT	2.34 ± 4.49	6.29 ± 5.08	0.063	3.55 ± 7.04	5.60 ± 3.72	0.391
FOG-Q	−2.34 ± 4.49	−6.29 ± 5.08	0.630	−2.55 ± 2.42	−2.5 ± 3.83	0.617
BBS	4.64 ± 3.32	2.92 ± 4.56	0.320	4.45 ± 4.41	2.92 ± 7.20	0.548
DGI^a^	0.91 ± 4.44	1.83 ± 2.37	0.901	0.63 ± 5.48	2.75 ± 3.31	0.221
MDS-UPDRS part 1	−4.73 ± 3.93	−2.25 ± 3.14	0.108	−3.72 ± 4.73	−3.00 ± 5.49	0.738
MDS-UPDRS part 2	−3.09 ± 3.62	1.67 ± 4.91	0.009^*^	−1.63 ± 3.61	1.75 ± 4.22	0.052
MDS-UPDRS part 3^a^	−23.27 ± 16.38	−19.08 ± 13.10	0.504	−4.64 ± 17.42	−9.75 ± 13.59	0.459
MDS-UPDRS part 4^a^	−1.09 ± 2.12	−2.92 ± 4.68	0.414	−0.82 ± 2.27	−3.33 ± 5.03	0.139

*T0, baseline; T1, immediately after intervention; T2, 4 weeks after intervention; 10MWT, 10m Walking Test; TUG, Timed Up and Go Test; FRT, Functional Reach Test; FOG-Q, Freezing of Gait Questionnaire; BBS, Berg Balance Scale; DGI, Dynamic Gait Index; MDS-UPDRS, Movement Disorder Society-sponsored revision of the Unified Parkinson's Disease Rating Scale,*

* *p < 0.05,*

a *Mann-Whitney test*.

### Gait

**Figure 2:**
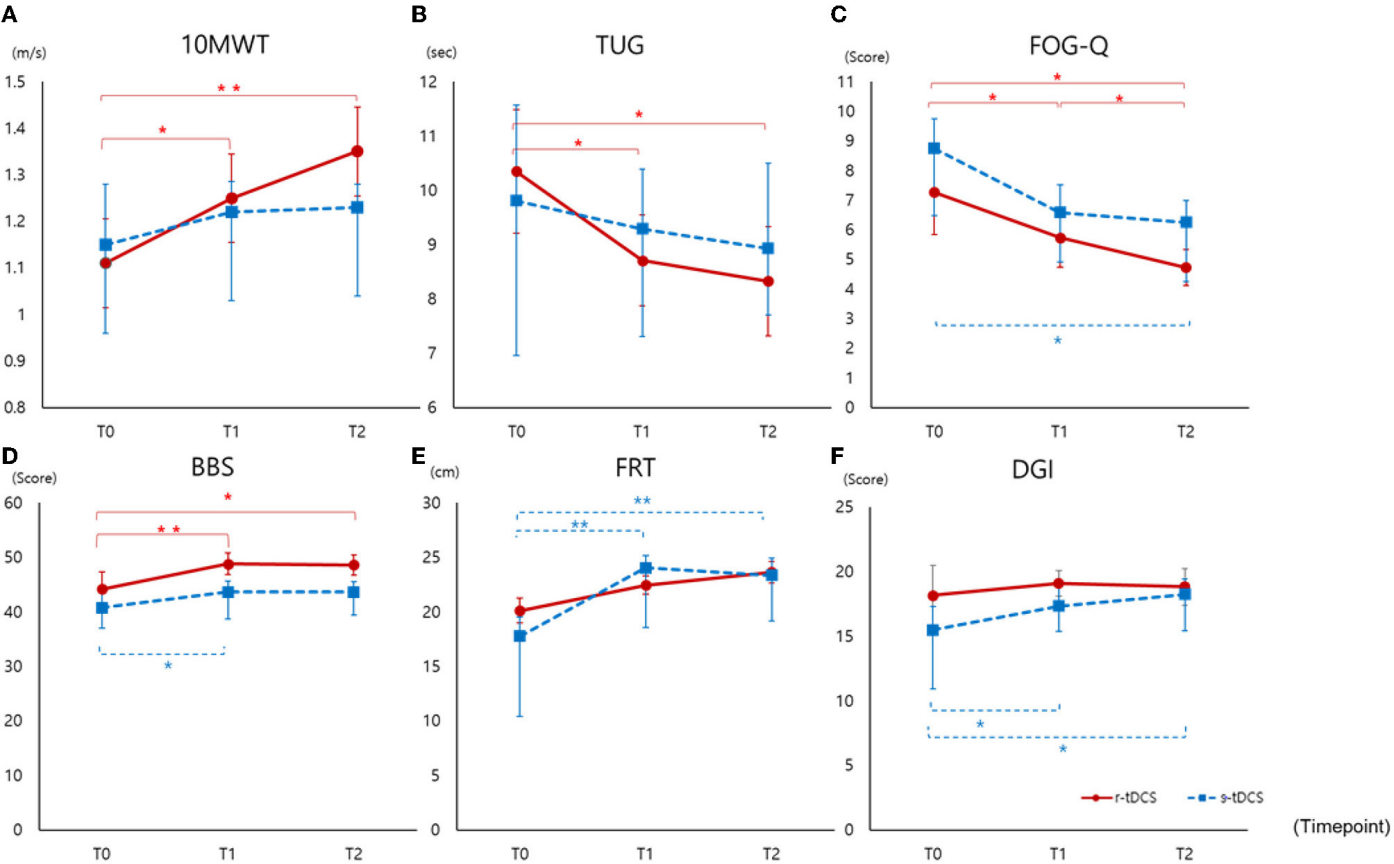
Outcomes measured before intervention (T0), immediately after intervention (T1), and at 4 weeks after completion of the intervention (T2) **(A)** 10MWT, **(B)** TUG, **(C)** FOG-Q, **(D)** BBS, **(E)** FRT, **(F)** DGI. 10MWT, 10-m walking test; TUG, Timed Up and Go Test; FRT, Functional Reach Test; FOG-Q, Freezing of Gait Questionnaire; BBS, Berg Balance Scale; DGI, Dynamic Gait Index; ^*^p < 0.05, ^**^ *p* < 0.005.

In the r-tDCS group, the increment of 10MWT was significant between T0 and T1 (*p* = 0.017) and between T0 and T2 (*p* = 0.002). On the other hand, in the s-tDCS group, there was no significant increase in 10MWT between T0 and T1 (*p* = 0.479) or between T0 and T2 (*p* = 0.341) (Figure 2).

Compared to sham intervention, the r-tDCS group showed decreased TUG at T1 (*p* = 0.014) and T2 (*p* = 0.005) compared to T0 (Figure 2). In the s-tDCS group, no significant changes were noted in TUG.

Freezing of gait improved significantly in both the groups, but the improvement was greater in the r-tDCS group than in the s-tDCS group at T2 (*p* = 0.006 vs. *p* = 0.045) compared to baseline. Comparing post-interventional performance with baseline in each group, the r-tDCS group showed early improvement at T1 (*p* = 0.007) compared to baseline (Figure 2). In terms of frequency of FOG, we analyzed FOG-Q #3 frequency. Both groups showed improvement at T1 and T2 compared to T0 (r-tDCS, T1 *p* = 0.002, T2 *p* < 0.001; s-tDCS T1 *p* = 0.005, T1 *p* = 0.010). There were no differences between the groups.

### Balance

Comparing r-tDCS and the s-tDCS groups, tDCS had no effect on improvement in balance with regard to DGI and FRT (Figure 2). The s-tDCS group showed improvement at T1 and T2 compared to T0 (DGI; T1 *p* = 0.021, T2 *p* = 0.015, FRT; *p* = 0.001, *p* ≤ 0.001). However, the improvement in balance, measured by BBS, in both the groups was greater in the r-tDCS than s-tDCS group at T1 (*p* = 0.001, *p* = 0.049). Comparing the 4-week post-interventional performance with baseline, balance amelioration remained significant in the r-tDCS group (*p* = 0.007).

### Movement Disorder Society-Sponsored Revision of the Unified Parkinson's Disease Rating Scale

**Figure 3:**
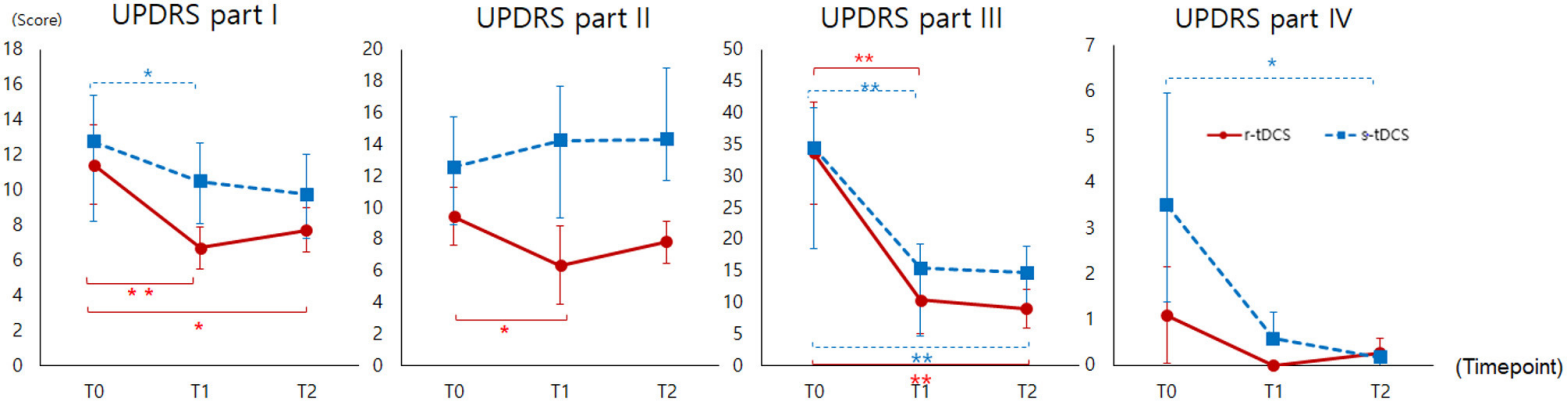
MDS-UPDRS (Part I to IV) measured before intervention (T0), immediately after intervention (T1), and at 4 weeks after completion of the intervention (T2). MDS-UPDRS, Movement Disorder Society-sponsored revision of the Unified Parkinson's Disease Rating Scale; *p < 0.05, ** *p* < 0.005.

Compared to the s-tDCS group, the r-tDCS group showed effects on non-motor and motor aspects of experiences of daily living. In motor examination, both the groups showed significant improvement. In motor complication, the experimental group showed trends toward decreasing complication, but they were not statistically significant (Figure 3). The MDS-UPDRS part I scores decreased in the r-tDCS group at T1 (*p* = 0.003) and T2 compared to T0 (*p* = 0.026). The s-tDCS group showed a significantly decreased MDS-UPDRS part I score at T1 compared to T0 (*p* = 0.030). In the MDS-UPDRS part II, the r-tDCS group showed a significantly decreased score at T1 compared to T0 (*p* = 0.018). In addition, the MDS-UPDRS part II demonstrated a significant interaction effect between the groups (Group^*^Time interaction, *p* = 0.042), which meant a greater improvement in the real group than the sham group. The MDS-UPDRS part III scores were decreased in both the groups at T1 (*p* = 0.001, *p* = 0.000) and T2 (*p* = 0.001, *p* = 0.000) compared to T0. The MDS-UPDRS part IV scores were significantly decreased only in the s-tDCS group at T2 compared to T0 (*p* = 0.042).

## Discussion

The major finding of this double-blind, randomized, sham-controlled study is that anodal stimulation of the M1_leg_ with multi-tDCS and simultaneous treadmill gait training were safe and effective in improving gait speed and other gait performances, including FOG and balance. The results of this study indicate that subjects with PD who undergo real tDCS and treadmill gait training improved in 10MWT, TUG, FOG, and BBS, but not in FRT or DGI. Furthermore, the s-tDCS group showed increases in motor aspects of activities of daily living (MDS-UPDRS part II) and motor examination (MDS-UPDRS part III). In addition, the s-tDCS showed improvement in FRT and DGI. In this section, we will discuss our findings in the light of previous studies and theories.

In previous trials, the therapeutic potential of tDCS for adjunctive treatment of PD has been assessed with promising results (24). However, this is the first study that comprehensively evaluates the therapeutic effect of multi-tDCS with treadmill gait training in PD in terms of qualitative gait improvement based on various clinical assessments. The r-tDCS group showed significant improvement in gait velocity, TUG test, and total FOG-Q scores. Our results showed that treadmill training with tDCS intervention can improve gait capacity (10MWT, TUG) and reduce FOG severity.

The change in mean velocity (T2–T0) of the r-tDCS group was 0.24 m/s, while that in the s-tDCS group (treadmill gait training only) was 0.07 m/s. According to previous research data from 633 patients with PD in the Cochrane database (2015), treadmill training enhanced the velocity and stride length of gait without affecting fall risk. Another study showed that treadmill training alone improved gait speed (mean differences = 0.09 m/s) (25). Considering that minimal detectable change (MDC) for 10MWT is 0.18 m/s for comfortable gait speed and 0.25 m/s for maximum gait speed (22), our findings appear reliable for the r-tDCS group. Quantitative differences between our findings and treadmill studies must be interpreted with caution because of the differences in sample size, test frequency, and duration of training. However, our results provide insight that such differences also might be caused by additional effects of tDCS.

Our results showed improvement of FOG-Q scores in both the groups at T2. This result supports the reported effect of treadmill gait training on improving the severity of FOG (26). Increased stride length and decreased step length variability due to treadmill gait training have been attributed to reduced FOG episodes (27). In our study, both the groups showed significant improvement at T1 and T2 compared to T0. Therefore, an additional effect of tDCS on the frequency of freezing gait was not verified in this study. Further study is required with a large number of participants to examine the effect of tDCS on FOG frequency. A previous pilot study (28) reported that application of anodal tDCS to the DLFPC improved TUG score in patients with PD and suggested that tDCS stimulation over the DLPFC might increase modulation of executive functions. We found that tDCS application to the M1_leg_ also improved TUG in patients with PD.

Compared to the r-tDCS group regarding improvement in BBS, the s-tDCS group (treadmill training only) showed improvement in BBS, FRT, and DGI. The treadmill can provide proprioceptive signals, triggering intact circuits and bypassing the defective pallidocortical circuit (29) to increase corticomotor excitability and induce motor learning (30). Patients with PD progressively lose flexibility and adaptability due to imbalances of neurotransmitters in the brain, resulting in trouble modulating gait parameters and short-step, narrow-based shuffling, and freezing with impaired internal gait rhythm. Treadmill activity is thought to cause cortical reorganization, especially in the supplementary motor area (31). This hypothesis is consistent with our findings in BBS and FRT.

Parkinson's disease is caused by the depletion of dopamine production in neurons in the basal ganglia (32). Basal ganglia control the ability to learn motor skills, such as walking and turning, by sending cues and sets to the cerebral cortex to help regulate the speed and amplitude of movements (33, 34). Dopamine release theory by tDCS can explain the significant r-tDCS improvement in BBS compared to that of s-tDCS. tDCS might cause a release of dopamine; widespread activation with anodal tDCS might release dopamine as the mechanism for improvement (35).

We designed the study with treadmill gait training and 10 sessions of multi-tDCS interventions and measured the outcomes 4 weeks later. We hypothesized that supplemental tDCS would have additional effects over gait training alone in PD and tried to verify whether the effect of tDCS persists after intervention. In 10MWT, TUG, FOG-Q, and BBS, tDCS showed effects late at T2 compared to baseline. As suggested by some authors (36), modification of the synaptic connections of the *N*-methyl-d-aspartate receptor (also known as the NMDA receptor or NMDAR) involved in long-term potentiation (LTP) can explain the long-lasting beneficial effects of tDCS. Some previous studies suggested that the efficacy of tDCS is enhanced when repeated, but the number of sessions for optimal response remains unknown (37). Further study is needed to determine optimal doses of tDCS intervention and the prolonged effects in PD.

Gait disturbances originate from various pathophysiological mechanisms, which might differ in their response to tDCS and its target regions. The mechanisms by which tDCS improves gait, balance, and other motor performance in PD are not known. The best evidence supporting the efficacy of brain stimulation was observed with DBS (4). DBS supposedly interferes with pathological activity and induces changes in activity (38, 39) and excitability (40) of the motor cortex, suggesting a possible mechanism that acts trans-synaptically along cortico–striato–thalamo–cortical circuits. According to a previous study (41), tDCS current can modulate membrane excitability and induce shifts in cortical excitability without rapid depolarization, directly generating action potentials. Anodal stimulation increases excitability and firing of active neurons and supposedly reverses decreased activity in motor and prefrontal cortices in PD. tDCS has been reported to enhance brain-derived neurotrophic factor (BDNF) secretion and tyrosine receptor kinase B (TrKB) activation *in vitro*, suggesting that it can improve motor learning through the promotion of synaptic plasticity (42).

Regarding the MDS-UPDRS, part I was improved in both the groups, but the increase was higher with r-tDCS. Part I mainly was developed to serve as an assessment of mentation, behavior, and mood. However, gait disorder in PD is multifactorial and might consist of different phenotypes, of which mood or behavior disorder is one expression. Part I of the MDS-UPDRS is limited to conclude an association between depression/mood problem and gait in our study. We speculated that tDCS increased extra-striatal dopamine release, similar to the mechanism of transcranial magnetic stimulation (TMS) (43), and it might affect non-motor performance improvement. While the r-tDCS group showed an increasing trend after intervention, the s-tDCS group experienced worsened score of the MDS-UPDRS part II. These contrasting results might be the effect of real tDCS intervention on motor aspects of daily living. Part III showed improvement in both the groups. In the MDS-UPDRS part IV, participants who received s-tDCS showed greater improvement than did the r-tDCS group. The average score of part IV in the s-tDCS group at baseline was higher than that in the r-tDCS group. In the same vein, the lack of improvement of DGI for the r-tDCS group is related to the better baseline function of the r-tDCS group compared to the s-tDCS group.

There are many study limitations that need to be addressed. Our small sample size and baseline functional differences preclude us from determining whether other demographic factors can account for differences in results. This is a pilot study with a small sample size, so further larger studies are needed to verify the clinical effect of tDCS on gait performance of patients with PD. Regarding MDC, only that of 10MWT was meaningful. Considering MDC for BBS is 5 points (22), the changes in both the groups fell short of the MDC. Neither TUG nor DGI in either group showed meaningful change (beyond random measurement error). Without a sufficient number of subjects, it was difficult to document large changes in MDC.

Compared to previous studies, we used a multichannel tDCS montage. This study is limited by the lack of conventional tDCS group. Comparison between multichannel tDCS and conventional tDCS would provide greater confidence in the outcomes of the study. There is no confirmed evidence of the superiority of multichannel tDCS over conventional tDCS. We hypothesized that targeting a distributed brain network of primary leg motor cortical areas rather than an isolated cortical region would facilitate better functional connectivity and motor learning in PD. One study (8) demonstrated that multi-tDCS could modulate ACC activity. Further study would be needed to confirm a neurophysiological change of multi-tDCS in PD. In addition, most patients with PD presented with mild to moderate disability without cognitive impairment, and so we were unable to determine whether our proposed intervention could be helpful to patients who have severe gait dysfunction. We used the K-MMSE to screen for mild cognitive impairment, despite knowing that the Montreal Cognitive Assessment (MoCA) is superior to the MMSE for detection of mild cognitive impairment or dementia in PD. Future studies using MoCA should be conducted to confirm the results with mild cognitive impairment. Though there were no group differences in sex (*p* = 0.47), the number of women was higher in r-tDCS group. It has been suggested that anodal stimulation causes a stronger excitability response in women than men (44). Sex ratio and its influence on the results should be considered in the future study.

## Conclusion

This study demonstrated that that multichannel tDCS combined with treadmill gait training is well-tolerated, safe, and effective in improving gait velocity (10MWT), gait capacity (TUG, FOG-Q), BBS, and motor aspects of the MDS-UPDRS. Our results of the primary outcome (gait velocity) indicate that tDCS with treadmill intervention is effective. The current study extends the knowledge of potential clinical therapeutic strategies. Since gait training rehabilitation is critical to treat gait disturbances of patients with PD, we suggest that clinical gait rehabilitation in patients with PD be combined with tDCS to enhance the effects. Also, as the concept of telerehabilitation is gaining popularity, which comprises home-based, professionally guided training sessions accessed v*ia* home-based devices such as video calls (45), the development of telerehabilitation with tDCS intervention could be a useful tool to improve functional outcomes.

## Data Availability Statement

The original contributions presented in the study are included in the article/Supplementary Material, further inquiries can be directed to the corresponding author/s.

## Ethics Statement

The studies involving human participants were reviewed and approved by the Institutional Review Board (IRB) of Samsung Medical Center, Seoul, South Korea. The patients/participants provided their written informed consent to participate in this study.

## Author Contributions

YN, JinK, S-HL, JL, SYS, WHC, JWC, and Y-HK designed the experiment. JinK, S-HL, and JihK collected the data. YN and JinK performed data and statistical analyses with assistance on approach and interpretation from SYS, JL, WHC, and Y-HK. YN wrote the manuscript. Y-HK critically evaluated the manuscript. All authors contributed to the article and approved the submitted version.

## Funding

This work was supported by the Samsung Medical Center and the National Research Foundation of Korea (NRF) grants funded by the Korean government [NRF-2020R1A2C3010304, NRF-2017M3A9G5083690].

## Conflict of Interest

The authors declare that the research was conducted in the absence of any commercial or financial relationships that could be construed as a potential conflict of interest.

## Publisher's Note

All claims expressed in this article are solely those of the authors and do not necessarily represent those of their affiliated organizations, or those of the publisher, the editors and the reviewers. Any product that may be evaluated in this article, or claim that may be made by its manufacturer, is not guaranteed or endorsed by the publisher.
