# Supplementary material for: Multichannel Transcranial Direct Current Stimulation Combined With Treadmill Gait Training in Patients With Parkinson's Disease: A Pilot Study
Source: Front Neurol. 2022 Mar 16;13:804206. doi: 10.3389/fneur.2022.804206 (PMC8966669; doi:10.3389/fneur.2022.804206)
Supplement: Supplementary file 1 [file Table_1.DOCX]

Supplementary table 1. Self-selected speed during gait training

| Treadmill gait speed, km/h  (mean±SD) | r-tDCS group  (n=11) | s-tDCS group  (*n* = 12) |
| --- | --- | --- |
| 1st intervention | 2.4±0.97 | 2.6±0.55 |
| 2nd intervention | 2.6±0.80 | 2.8±0.38 |
| 3rd intervention | 2.8±0.69 | 2.8±0.41 |
| 4th intervention | 2.8±0.69 | 3.0±0.39 |
| 5th intervention | 2.9±0.68 | 3.1±0.74 |
| 6th intervention | 3.0±0.66 | 3.2±0.53 |
| 7th intervention | 3.2±0.64 | 3.2±0.46 |
| 8th intervention | 3.2±0.63 | 3.1±0.44 |
| 9th intervention | 3.2±0.65 | 3.2±0.46 |
| 10th intervention | 3.2±0.69 | 3.0±0.51 |
